# Intratracheal Ovalbumin Administration Induces Colitis Through the IFN-γ Pathway in Mice

**DOI:** 10.3389/fimmu.2019.00530

**Published:** 2019-03-21

**Authors:** Kyoung-Hwa Jung, Dasom Shin, Sejun Kim, Daeun Min, Woogyeong Kim, Jinju Kim, Gihyun Lee, Hyunsu Bae

**Affiliations:** ^1^Department of Physiology, College of Korean Medicine, Kyung Hee University, Seoul, South Korea; ^2^Department of Korean Physiology, College of Pharmacy, Kyung Hee University, Seoul, South Korea; ^3^College of Korean Medicine, Dongshin University, Naju, South Korea

**Keywords:** allergy and immunology, colitis, IFN-γ, ovalbumin, Th1 cells

## Abstract

Recent studies have reported an increased incidence of inflammatory bowel disease (IBD) in patients with pulmonary diseases. Despite clinical and epidemiological studies of the interplay between colitis and asthma, the diseases' related underlying mechanisms remain unclear. In this study, we evaluated the development of colitis in a model of allergic airway inflammation. We revealed that intratracheal chronic ovalbumin (OVA) exposure induces colitis and allergic airway inflammation. Interestingly, induction of colitis was largely regulated by Th1, rather than Th2 responses, whereas allergic airway inflammation was primarily mediated by Th2 responses. Experiments in *Tbx21* (T-bet) and *Ifng* (IFN-γ) deficient mice have confirmed that IFN-γ is a major mediator involved in OVA-induced colitis. These findings broaden current understanding of allergen induced colitis pathology and could play a role in the development of novel clinical treatment strategies for asthmatic patients who are at risk of developing colitis.

## Introduction

Cytokines play a crucial role in the pathogenesis of inflammatory bowel diseases (IBD), such as Crohn's disease, regulating diverse aspects of the inflammatory reaction (1). IFN-γ is a primary proinflammatory cytokine involved in Crohn's disease pathogenesis (2). IFN-γ is critical in the regulation of multiple immune functions, such as antigen presentation, cellular proliferation, leukocyte trafficking, microbicide effector activation, and pathogen recognition (3). However, overproduction of IFN-γ is implicated in many gastrointestinal disorders, including Crohn's disease, celiac disease, and autoimmune gastritis (4–6), and also plays a role in pulmonary inflammation (7).

Several recent studies have reported a link between inflammatory bowel disease and pulmonary inflammation (8–11) Incidence of IBD is significantly increased among patients with lung disorders (12). Despite these epidemiological and clinical observations, few experimental studies have investigated the role of airway allergic responses in the development of IBD. To reveal the mechanisms underlying the effects of allergic airway inflammation on IBD pathogenesis, we hypothesized that intratracheal ovalbumin (OVA) exposure would induce inflammation in both the lung and the colon, as it is well-known that OVA exposure causes Th2- and Th1-mediated airway inflammation responses (13). Notably, we observed that intratracheal OVA exposure is sufficient to induce colitis mediated by Th1 responses, not Th2 responses. Based on these results, we investigated the role of Th1 responses in OVA exposure induced colitis using T-bet or IFN-γ deficient mice. In this study, we demonstrate that IFN-γ plays a key role in colitis induced by intratracheal exposure to OVA.

## Materials and Methods

### Animals

This study was approved by the Kyung Hee University animal care and use committee. All of the experiments were performed in accordance with the approved animal protocols and guidelines established by Kyung Hee University [KHUASP(SE)-17-039]. Female 6- to 8-week-old C57BL/6 mice were used for experiments. Ancestors of WT and IFN-γ knockout (Ifng^−/−^, B6.129S7-Ifngtm1Ts/J) mice used for this study were purchased from the Jackson Laboratory (Bar Harbor, ME, USA). Ancestor of T-bet knockout (Tbet^−/−^) mice were provided by Dr. Laurie Glimcher (Dana-Farber Cancer Institute). All mice have been bred in the same room for at least 10 generations, eating the same food to exclude the influence of microbiota. Sex-matched littermate controls were used in all experiments. All of the mice were housed under specific pathogen-free conditions in the Animal Barrier Facility.

### Animal Models of Allergic Airway Disease and Colitis

**Figure 1:**
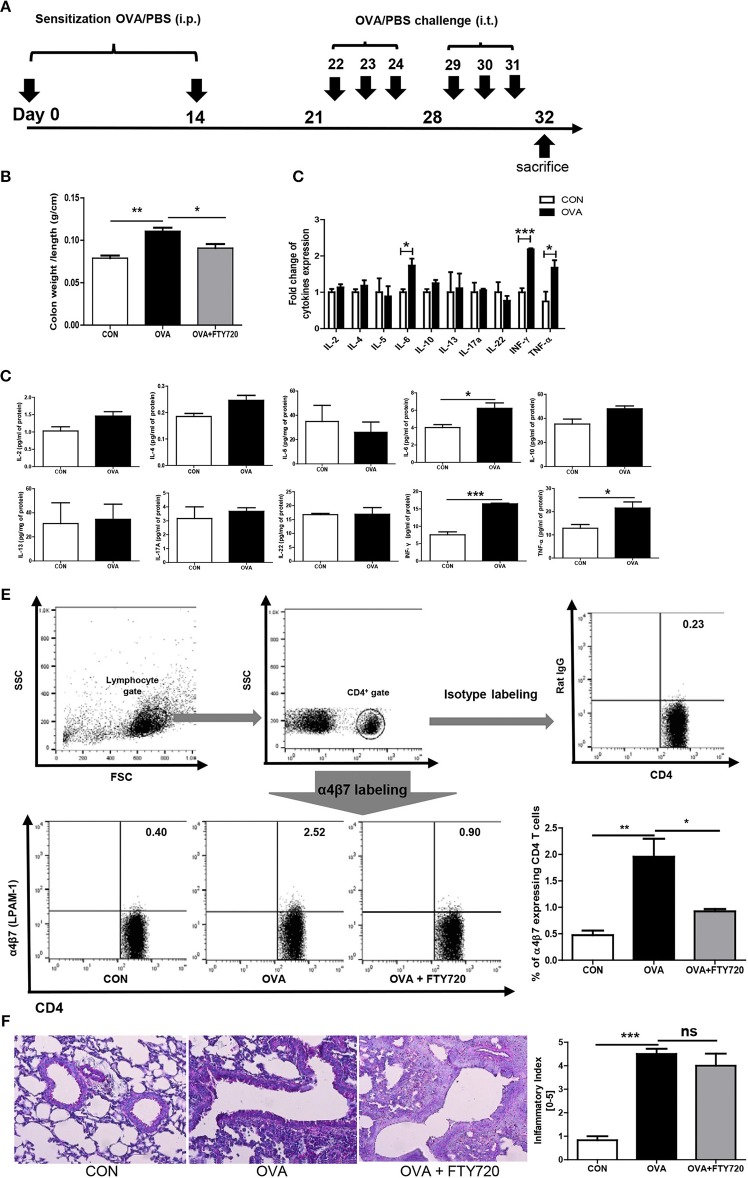
Induction of OVA allergen related colitis. **(A)** Mice (*n* = 16) were sensitized via i.p. injection of OVA mixed with aluminum hydroxide on days 0 and 14. Sensitized mice were intratracheally challenged with 1% OVA, six times between days 22 and 31. Normal control (CON) mice were sensitized and challenged with PBS alone. **(B)** The ration between the colon weight and length are shown. **(C,D)** The expression of cytokines in the colon after OVA exposure is shown. **(E)** The percentage of α4β7 expressing CD4 cells are shown. **(F)** A representative image of lung histology after OVA and FTY720 treatment and quantified score of severity of allergic airway inflammation are shown. Data are presented as the mean ± SEM and the *p-*value was estimated by unpaired *t*-test (*P < 0.05, **P < 0.01, and ***P < 0.001).

WT (*n* = 16), Tbet^−/−^ (*n* = 15) and Ifng^−/−^ (*n* = 16) mice were sensitized on days 0 and 14 by intraperitoneal (i.p.) injection of 5 mg of OVA (Sigma-Aldrich) emulsified in 1 mg of aluminum hydroxide. Subsequently, sensitized mice were intratracheally applied with 0.5 μg of OVA in 50 μl of PBS on days 22, 23, 24, 29, 30, and 31 after initial OVA exposure (Figure 1A). Twenty-four h after the last challenge, mice were sacrificed (14, 15).

### Cytokine Measurement

Colonic tissues and lung tissues were homogenized with a T-PER tissue lysis buffer (Thermo Fisher Scientific Inc., Waltham, MA, USA) containing a protease inhibitor cocktail (Roche Diagnostics, Mannheim, Germany) using a Precellys 24 homogenizer (Bertin Technologies, France) and centrifuged at 13,000 rpm for 15 min at 4°C. Colon cytokine levels in the homogenates were measured using a murine Th1, 2 and 17 Cytometric Bead Array (CBA kit; BD sciences, San Diego, CA, USA) or ELISA kits [IL-4, IL-5, IL-6, IFN-γ, TNF-α from BD sciences (16); IL-2, IL-13, IL-17, IL-22 from R&D, Minneapolis, MN, USA (17)]. Lung cytokine levels in the homogenates were measured using ELISA kits [IL-4, IL-5, IL-6, IFN-γ, TNF-α from BD sciences (16); IL-13 from R&D, Minneapolis, MN, USA (17)]. Total protein concentrations were characterized with a Bio-Rad protein assay (BioRad, Hercules, CA, USA) read on a microplate reader (SOFT max PRO software, Sunnyvale, CA, USA).

### Histological Analysis

Longitudinally divided rolled-up parts of the colon were used for histological analysis. Harvested lung tissues were directly fixed in 10% neutral-buffered formalin overnight at 4°C. The tissue samples were dehydrated and then paraffin-embedded (cut into colon: 10-μm-thick; lung: 4-μm-thick sections) using a rotary microtome. The four sections of colon and lung were stained with hematoxylin & eosin (H&E), respectively. Image of colon and lung sections were captured using an Olympus BX51 microscope (Olympus, Tokyo, Japan) equipped with a DP71 digital camera (Olympus, Tokyo, Japan) under x200 magnifications. Colon histological samples were scored by blinded investigators as described by Erben et al. (18). Colon samples were scored between 0 and 5, with the score calculated using the following criteria: 0 = normal colon mucosa with intact epithelium; 1 = scattered inflammatory cell infiltrates in the mucosa; 2 = diffuse mucosal infiltrates without submucosal spreading and an intact epithelial layer; 3 = moderate infiltration of inflammatory cells into the mucosa and submucosa with epithelial hyperplasia and goblet cell loss; 4 = marked infiltration of inflammatory cells into the mucosa and submucosa accompanied by crypt abscesses and loss of goblet cells and crypts; 5 = marked infiltration of inflammatory cells into the mucosa and submucosa accompanied by crypt loss and hemorrhage. Lung samples were scored as: 0 = normal; 1 = very mild; 2 = mild; 3 = moderate; 4 = marked; or 5 = severe inflammation as described by Tate et al. (19).

### Differential (Diff) Cells Counts in BAL Fluid

To collect BAL fluid, ice-cold PBS (1 ml) was infused into the lungs and withdrawn via tracheal cannulation three times, respectively (harvested BAL fluid volume 2.0-2.5 ml). The harvested BAL fluid was centrifuged at 1,300 rpm for 10 min at 4°C; supernatants were removed and the BAL cellular pellet was re-suspended in 1 ml of ice-cold PBS. Next, BAL cells were adhered to glass sides using a Cytospin (Sandon, Waltham, MA, USA) and stained with Diff-Quick. The stained BAL cells were dried and mounted with a non-aqueous medium (Diamount, Diapath. Martinengo, BG, Italy). The BAL cells were counted under a light microscope following the method we previously reported (15).

### FTY720 Treatment and α4β7 Measurement

WT mice (*n* = 16) were sensitized on days 0 and 14 by i.p. injection of OVA (Sigma-Aldrich; 5 mg) emulsified in aluminum hydroxide (1 mg). FTY720 (Sigma-Aldrich, St. Louis, Missouri, USA; 0.8 mg/kg) was administered intraperitoneally 30 min prior to intratracheal OVA administration on days 22, 23, 24, 39, 30, and 31 (20, 21). The spleens were disrupted over a wire mesh screen in 2 ml PBS. The spleen supernatant was collected in a 15 ml tube and centrifuged for 10 min at 300 g. The supernatant was removed and BD Pharm Lyse™ lysing solution (BD sciences, San Diego, CA, USA) added to the red blood cells for 5 min. The cells were washed with 9 ml PBS and centrifuged for 10 min at 300 g to give a single cell suspension ready for staining. To measure gut homing integrin α4β7, splenocytes were incubated with CD4-APC and α4β7-PE antibodies (e-Bioscience, San Diego, CA, USA). Samples were first gated for lymphocytes, and then for CD4 positive cells; finally, to analyze α4β7 in CD4 cells, CD4 positive and α4β7 positive cells were gated. All sample data were acquired using FACSCalibur (BD sciences, San Diego, CA, USA) and analyzed using FlowJo software (Tree Star Inc., Ashland, OR, USA).

### Isolation of Colonic Lamina Propria Leukocytes

To isolate the colonic lamina propria, entire colons were cut longitudinally and washed to remove feces and debris. Sections of colon were incubated in HBSS containing 5 Mm EDTA and 2% FBS in a shaking incubator at 37°C for 25 min. After washing three times in PBS to remove EDTA, the colon sections were finely minced and incubated in digestion media containing HBSS, 1 mg/mL collagenase VIII (Sigma-Aldrich), 0.01 mg/mL DNase (Roche), and 1M 2βME (Sigma-Aldrich) in a shaking incubator at 37°C for 45 min. Following collagenase digestion, the medium containing the mononuclear cells was collected, filtered, and centrifuged at 300 g for 10 min.

### Statistical Analysis

The statistical analyses of the data were conducted using Prism 5 software (GraphPad Software Inc., La Jolla, CA, USA). Date represented as the means ± SEM. The statistical significance (*p* < 0.05) was assessed using an unpaired *t*-test.

## Results

### Chronic Intratracheal OVA Exposure Induces Colitis

Chronic OVA exposure is commonly used to establish allergic airway inflammation in mice. To determine if chronic OVA exposure causes development of colitis, we challenged mice with PBS or OVA following an established protocol for a murine model of asthma (Figure 1A).

As shown in Figure 1B, colonic weight/length was significantly increased in the OVA group (OVA challenged mice) compared to the control group (PBS challenged mice). To elucidate the immunological mechanisms of allergic airway inflammation-induced colitis, we analyzed cytokine expression in colonic tissue. Surprisingly, levels of IFN-γ were highly elevated; other proinflammatory cytokines were elevated, including TNF- α and IL-6, suggesting that OVA-induced colitis is mediated through Th1 responses. Interestingly, there was no increase in the level of cytokines, including IL-4, IL-5, IL-13, IL-17, and IL-22, produced by Th2 and Th17 cells in the colon (Figures 1C,D), but there was in the lungs (**Figure 4**).

### FTY720 Treatment Inhibits Colonic Inflammation Induced by Chronic Intratracheal OVA

Researchers are currently trying to identify the mechanisms of interaction between the gastrointestinal and respiratory systems (22). We hypothesized that circulating immune cells could mediate cross-talk between the lungs and colon, specifically, that chronic intratracheal OVA-induced colitis could be mediated by circulating activated CD4 T cells. Activated CD4 T cells in the intrathoracic lymph nodes can migrate to other organs and cause a pathological response. To determine whether our hypothesis was correct, we first measured α4β7 integrin expression in circulating T cells. α4β7 is important in the homing of T cells to intestinal sites and is required for the induction of chronic colitis (23). The level of α4β7-expressing CD4 T cells was increased by chronic intratracheal OVA exposure in the blood and spleen, indicating that these OVA-stimulated cells may migrate to the colon and cause inflammation (Figure 1E). In addition, i.p. treatment with the immunomodulator FTY720, which interferes with the trafficking of cells between lymphoid organs and the blood (24) and causes lymphopenia (25) inhibited the progress of colitis and increased the colon weight/length ratio (Figure 1B), but did not improve lung inflammation (Figure 1F). This was accompanied by decreased α4β7 expression in CD4 T cells (Figure 1E). These data suggest that circulating immune cells mediate chronic intratracheal OVA-induced colitis. FTY720 treatment also led to lymphopenia and affected both the percentage of total lymphocytes (69.98 ± 1.182% to 44.77 ± 1.320, *P* < 0.001) and CD4 cells (26.40 ± 1.895% to 13.70 ± 0.6234, *P* < 0.001) in the spleen.

### T-Bet and IFN-γ Are Crucial for Chronic Intratracheal OVA Exposure Induced Colitis

**Figure 2:**
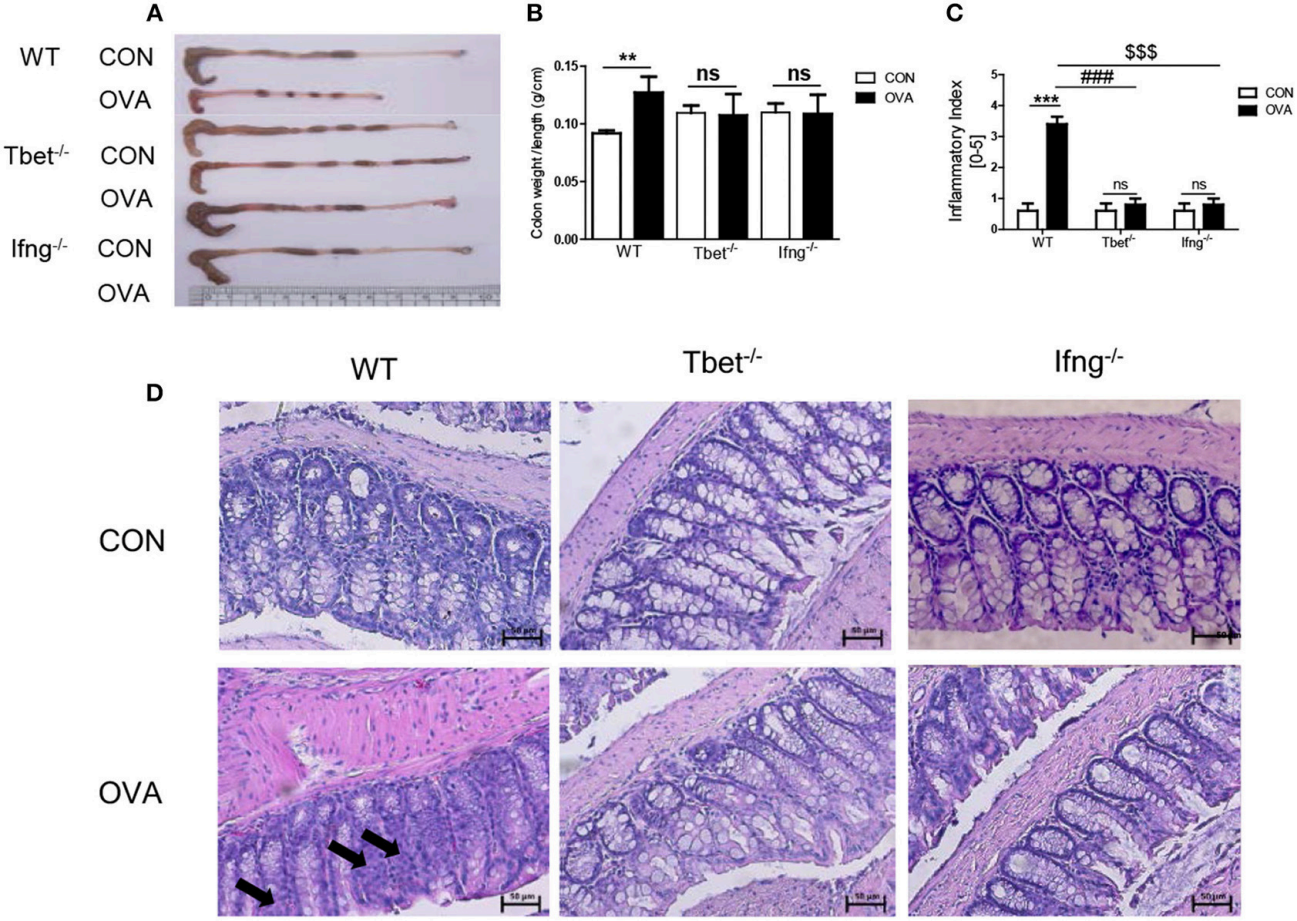
Pathological changes in the colon tissues after intratracheal OVA exposure. **(A)** Representative photos of whole colon in OVA induced colitis. **(B)** A graph indicating colon weight/length in WT (*n* = 16), Tbet^−/−^ (*n* = 15), and Ifng^−/−^ (*n* = 16) mice. **(C)** The severity of colitis was scored as described in the material and method. CON: only PBS injected mice; OVA: OVA injected mice. **(D)** Representative image of colon histology. Arrows indicate inflammation sites. To observe the histopathological changes after exposure to OVA, colonic tissues were stained via H&E (magnification 200x). Data are presented as the mean ± SEM and *p*-value was estimated using an unpaired *t*-test (**P < 0.01, ***P < 0.001, ^*###*^*P* < 0.001 WT OVA vs. Tbet^−/−^ OVA, ^*$$$*^*P* < 0.001 WT OVA vs. Ifng^−/−^ OVA, and ns, not significant).

**Figure 3:**
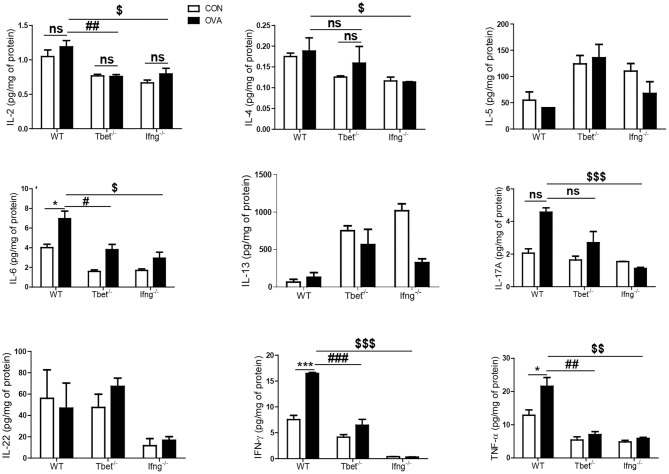
Cytokines alteration by intratracheal OVA exposure in colon of WT, Tbet^−/−^, and Ifng^−/−^ mice. The levels of IL-2, IL-4, IL-5, IL-6, IL-13, IL-17A, IL-22, IFN-γ, and TNF-α were measured in the colonic homogenates from WT (*n* = 16), Tbet^−/−^ (*n* = 15) and Ifng^−/−^ (*n* = 16) mice. Data are presented as the mean ± SEM and *p*-value was estimated by unpaired *t*-test (*P < 0.05, ***P < 0.001, ^#^*P* < 0.05, ^##^*P* < 0.01, ^###^*P* < 0.001, ^$^*P* < 0.05, ^$$^*P* < 0.01, ^$$$^*P* < 0.001, and ns, not significant).

To confirm that induction of colitis after chronic OVA exposure is mediated via Th1 responses, we challenged Tbet^−/−^ and Ifng^−/−^ mice with OVA. Chronic OVA exposure did not alter colon length in Tbet^−/−^ or Ifng^−/−^ mice (Figures 2A,B). Histological analysis confirmed that chronic OVA exposure induces colitis mediated through Th1 responses. OVA-challenged WT mice exhibited severe inflammation and damage in the colon. Colonic tissue from OVA-exposed mice showed thickened muscularis and increased inflammatory cell infiltration into the mucosa, whereas OVA challenge in Tbet^−/−^and Ifng^−/−^ mice did not result in histological damage (Figures 2C,D). Additionally, T-bet or IFN-γ deficiency restored the OVA-induced overproduction of inflammatory cytokines, including TNF-α, IL-6, and INF-γ (Figure 3).

### T-Bet and IFN-γ Are not Critical for OVA Exposure Induced Allergic Airway Inflammation

**Figure 4:**
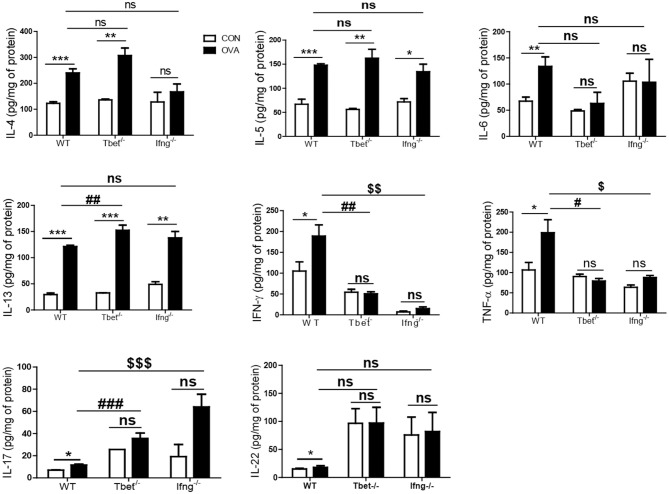
Cytokines alteration by intratracheal OVA exposure in the lung of WT, Tbet^−/−^, and Ifng^−/−^ mice. The levels of IL-4, IL-5, IL-6, IL-13, IL-17A, IL-22, IFN-γ, and TNF-α were measured in the lung homogenates from WT (*n* = 16), Tbet^−/−^ (*n* = 15) and Ifng^−/−^ (*n* = 16) mice. Data are presented as the mean ± SEM and *p*-value was estimated by unpaired *t*-test (*P < 0.05, **P < 0.01, ***P < 0.001, ^#^*P* < 0.05, ^*##*^*P* < 0.01, ^*###*^*P* < 0.001 WT OVA vs. Tbet^−/−^ OVA, ^$^*P* < 0.05, ^$$^*P* < 0.01, ^$$$^*P* < 0.001 WT OVA vs. Ifng^−/−^ OVA, and ns, not significant).

**Figure 5:**
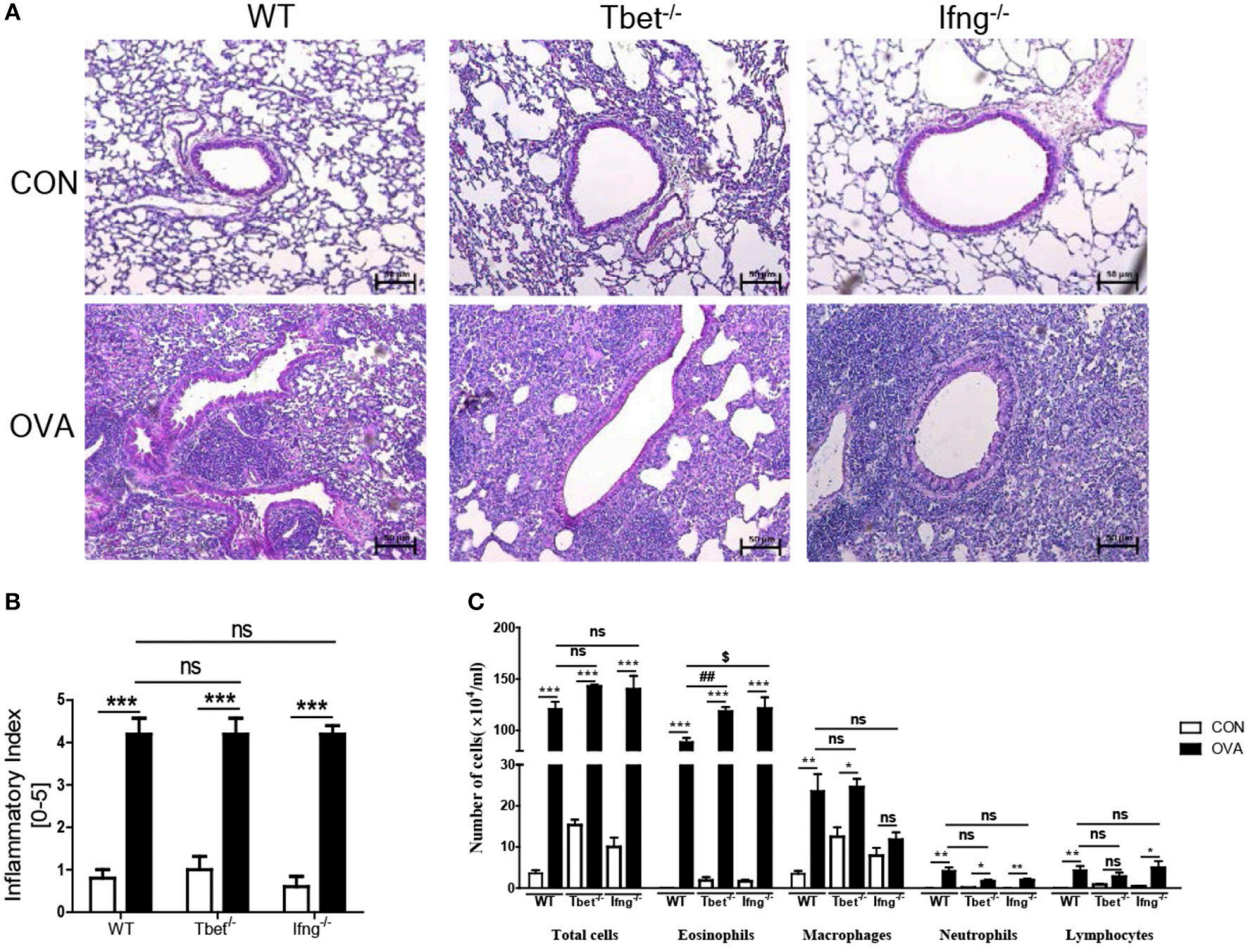
Pathological changes in the lung after intratracheal OVA exposure. **(A)** Representative image of lung histology. **(B)** Assessment of histological changes of severity of allergic airway inflammation was quantified using a scoring system. **(C)** Inflammatory cell profiles in the BAL fluid after OVA exposure. WT (*n* = 16), Tbet^−/−^ (*n* = 15) and Ifng^−/−^ (*n* = 16) mice. Data are presented as the mean ± SEM and *p*-value was estimated by unpaired *t*-test (*P < 0.05, **P < 0.01, ***P < 0.001, ^##^*P* < 0.01, ^$^*P* < 0.05, and ns, not significant).

It is well-known that the allergic airway inflammation is dependent on Th2 responses and independent of Th1 responses (26). Considering that development of colitis after chronic exposure to OVA was dependent on Th1 responses, we examined if Th1 responses play a role in OVA-induced allergic airway inflammation. As shown Figure 4, OVA exposure significantly increased the production of Th1, Th2, and Th17 cytokines in the lungs of WT mice compared with the control group. Tbet^−/−^ and Ifng^−/−^ mice did not show increased production of Th1 or Th17 cytokines (IFN-γ, TNF-α, IL-6, IL-17, and IL-22), however they showed significantly increased production of Th2 cytokines (IL-4, IL-5, and IL-13 in Tbet^−/−^ mice; IL-5 and IL-13 in Ifng^−/−^ mice). Notably, our data showed that expressions of IFN-γ, TNF-α and IL-6 were significantly increased in the lung of OVA-challenged WT mice. Tbet^−/−^ and Ifng^−/−^ mice did exhibit alterations in Th1 response, as expected. Altogether, the data suggest that OVA exposure leads to Th1, Th2, and Th17 responses in the lungs of C57BL/6J mice and that Th1 responses are not critical for OVA-induced allergic airway inflammation. In addition, OVA exposure resulted in the significant infiltration of inflammatory cells into the lung parenchyma in all WT, Tbet^−/−^, and Ifng^−/−^ mice. Tbet^−/−^ and Ifng^−/−^ mice also had elevated inflammatory cell accumulation around the bronchi following OVA challenge, similar to the observations made in WT mice (Figure 5). Therefore, these data suggest that OVA-induced allergic airway inflammation is not dependent on Th1 responses, as opposed to OVA-induced colitis.

## Discussion

Recently, the link between colitis and pulmonary diseases has been discussed in the literature. Several cross-sectional studies reported an increased risk of colitis in patients with allergic disorders (12, 22, 27). While it is well-known that pathogen exposure of the lungs leads to development of allergic responses in the lung, the effects on a remote site such as in the colon still remained uncertain (22). We demonstrated that cigarette smoke induced colitis via IFN- γ (28) whilst another group showed that impaired gas exchange associated with cigarette smoke caused systemic and intestinal ischemia, driving angiogenesis and gastrointestinal tract epithelial barrier dysfunction, and resulting in the increased risk and severity of Crohn's disease (29). This study confirmed that colitis is mediated by the Th1 response, in particular IFN-γ-producing CD4 T cells. Based on our previous study, we confirmed the induction of colitis by exposing the airway to an allergen (OVA) and clarified the mechanism of this induction.

Our experiments demonstrate that the Th1 cell-specific transcription factor T-bet is robustly involved in OVA-induced colitis. Intratracheal OVA-induced colitis resulted in production of T-bet mediated cytokines, including TNF-α, IL-6, and IFN-γ in normal mice, while OVA exposure of T-bet deficient mice failed to result in induction of colitis. Furthermore, experimentation using IFN-γ deficient mice confirmed that IFN-γ mediates airway allergic response induced colitis. In fact, our results support the general notion that pathogenesis of Crohn's disease is predominantly associated with a Th1 response, but not with Th2 response (30). As IFN-γ plays a necessary role in the development of colitis (31), anti-IFN-γ has been applied as a targeted therapy in the treatment of Crohn's disease (32). Recently, involvement of the Th17 response in the pathogenesis of colitis has been reported (33). In our experiment, intratracheal administration of OVA caused a stronger Th17 response in the lungs of T-bet deficient mice than in WT mice. This agrees with previous studies, which have demonstrated that T-bet suppresses Th17 differentiation, consequently leading to higher levels of IL-17A in T-bet KO mice than in WT mice (34). However, intratracheal administration of OVA did not alter IL-17A levels in the colons of WT, T-bet deficient, or IFN-γ deficient mice. The IFN-γ pathway is not the only mechanism linking inflammatory bowel disease and pulmonary inflammation; a recent study demonstrated the role of macrophages in cigarette smoke-induced colitis (35). Indeed, we also observed an increase in the number of macrophages in the colonic laminar propria after OVA sensitization (Supplementary Figure 1), thus detailed mechanisms need to be identified in subsequent studies.

FTY720 treatment can influence the development of asthma, however the effects may differ depending on the mode of administration. Sawicka et al. used the adoptive transfer model for Th1 and Th2 cells in asthma development. In their model, FTY720 inhibited the migration of Th1 and Th2 cells to the lungs, resulting in attenuated asthma as FTY720 also prevents the migration of lymphocytes to inflammatory sites (13). Idzko et al. applied FTY720 locally via inhalation, which inhibited the migration of lung DCs to the mediastinal lymph nodes and blocked the formation of Th2 cells in the lymph nodes (36). In our experiments, we i.p. injected FTY720 to block the migration of inflammatory cells to the colon. Systemic FTY720 treatment may have less of an effect on the primary site of inflammation than local treatment, and may explain why our FTY720 treatment did not affect the development of asthma.

This initial study indicates a promising future of potential therapeutic discovery and subsequent treatment of colitis in patients with allergic airway inflammation, including asthma. We found a circulating CD4 T cell mediated mechanism for interaction between the gastrointestinal and respiratory systems. It, however is still remained a lack of understanding of the cross-talk between lung and colon. Further studies are needed to broaden our knowledge of the cross-talk between lung and colon.

## Author Contributions

HB and JK conceived and designed the experiments. K-HJ and DS performed the majority of experiments. WK, DM, and SK contributed to the acquisition of data. GL wrote the paper. All authors read and approved the final manuscript.

### Conflict of Interest Statement

The authors declare that the research was conducted in the absence of any commercial or financial relationships that could be construed as a potential conflict of interest.

## Supplementary Material

Supplementary Figure 1 Colonic lamina propria and spleen were isolated and single cell suspensions prepared. After incubation with CD45-FITC, CD11b-PE, and F4/80-APC antibodies, the cells were analyzed using flow cytometry. Upper panel shows the percentage of macrophages in colonic lamina propria and the lower panel macrophages in spleen.
