# Supplementary figures and images for: Intratracheal Ovalbumin Administration Induces Colitis Through the IFN-γ Pathway in Mice
Source: Front Immunol. 2019 Mar 21;10:530. doi: 10.3389/fimmu.2019.00530 (PMC6437076; doi:10.3389/fimmu.2019.00530)

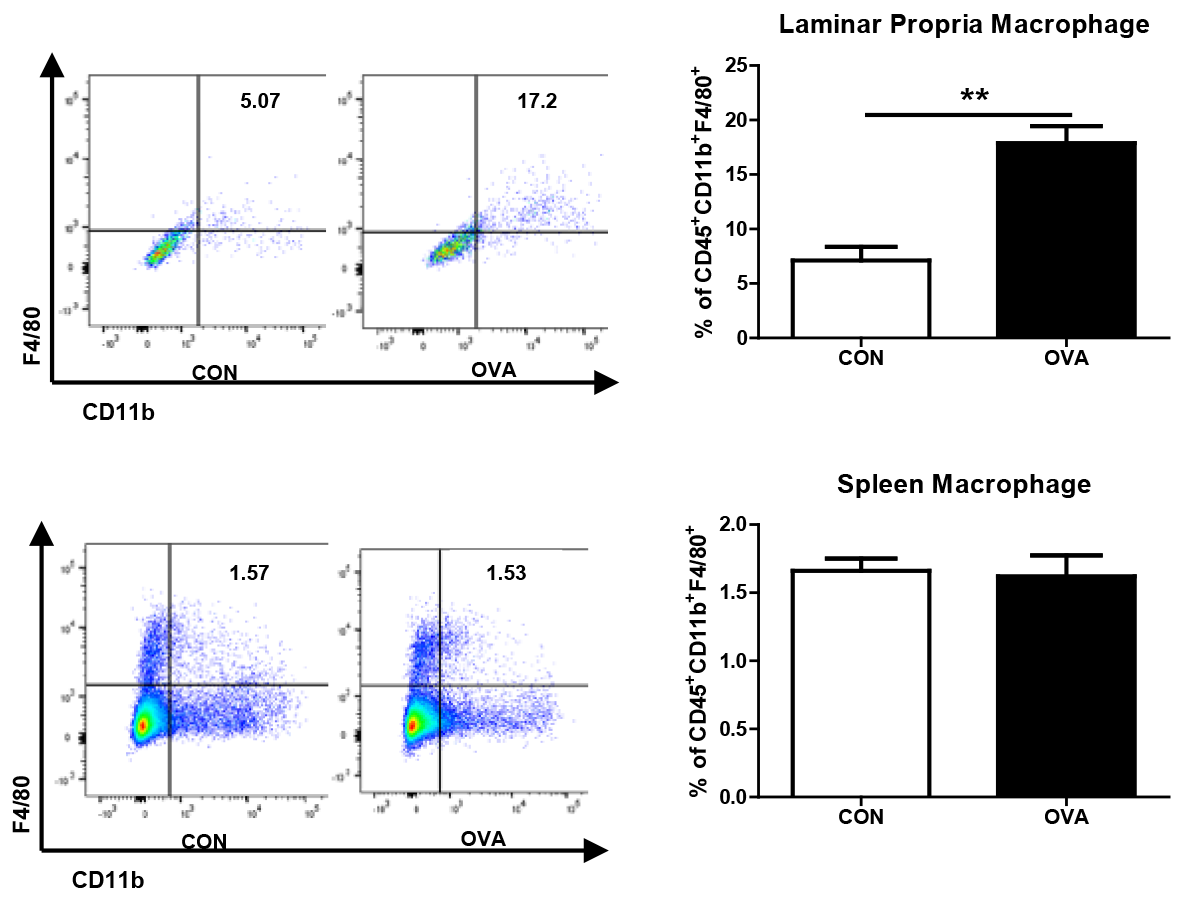

Supplement: Supplementary Figure 1 — Colonic lamina propria and spleen were isolated and single cell suspensions prepared. After incubation with CD45-FITC, CD11b-PE, and F4/80-APC antibodies, the cells were analyzed using flow cytometry. Upper panel shows the percentage of macrophages in colonic lamina propria and the lower panel macrophages in spleen. [file Image_1.TIF]
